# Presenilin-1 regulates the constitutive turnover of the fibronectin matrix in endothelial cells

**DOI:** 10.1186/1471-2091-13-28

**Published:** 2012-12-21

**Authors:** Rita De Gasperi, Miguel A Gama Sosa, Gregory A Elder

**Affiliations:** 1 Research and Development, James J. Peters Department of Veterans Affairs Medical Center, Bronx, NY, 10468, USA; 2 Neurology Service, James J. Peters Department of Veterans Affairs Medical Center, Bronx, NY, 10468, USA; 3 Department of Psychiatry, Icahn School of Medicine at Mount Sinai, New York, NY, 10029, USA; 4 Department of Neurology, Icahn School of Medicine at Mount Sinai, New York, NY, 10029, USA; 5 Friedman Brain Institute, Icahn School of Medicine at Mount Sinai, New York, NY, 10029, USA

**Keywords:** Endothelial cells, Extracellular matrix, Fibronectin, Presenilin-1, Vascular development

## Abstract

**Background:**

Presenilin-1 (PS1) is a transmembrane protein first discovered because of its association with familial Alzheimer’s disease. Mice with null mutations in PS1 die shortly after birth exhibiting multiple CNS and non-CNS abnormalities. One of the most prominent features in the brains of PS1−/− embryos is a vascular dysgenesis that leads to multiple intracerebral hemorrhages. The molecular and cellular basis for the vascular dysgenesis in PS1−/− mice remains incompletely understood. Because the extracellular matrix plays key roles in vascular development we hypothesized that an abnormal extracellular matrix might be present in endothelial cells lacking PS1 and examined whether the lack of PS1 affects expression of fibronectin a component of the extracellular matrix known to be essential for vascular development.

**Results:**

We report that primary as well as continuously passaged PS1−/− endothelial cells contain more fibronectin than wild type cells and that the excess fibronectin in PS1−/− endothelial cells is incorporated into a fibrillar network. Supporting the *in vivo* relevance of this observation fibronectin expression was increased in microvascular preparations isolated from E14.5 to E18.5 PS1−/− embryonic brain. Reintroduction of PS1 into PS1−/− endothelial cells led to a progressive decrease in fibronectin levels showing that the increased fibronectin in PS1−/− endothelial cells was due to loss of PS1. Increases in fibronectin protein in PS1−/− endothelial cells could not be explained by increased levels of fibronectin RNA nor based on metabolic labeling studies by increased protein synthesis. Rather we show based on the rate of turnover of exogenously added biotinylated fibronectin that increased fibronectin in PS1−/− endothelial cells results from a slower degradation of the fibronectin fibrillar matrix on the cell surface.

**Conclusions:**

These studies show that PS1 regulates the constitutive turnover of the fibronectin matrix in endothelial cells. These studies provide molecular clues that may help to explain the origin of the vascular dysgenesis that develops in PS1−/− embryonic mice.

## Background

Presenilin-1 is a polytopic transmembrane protein that was first discovered because of its association with familial Alzheimer’s disease [1,2]. PS1 is highly conserved in evolution having homologues in organisms as distant as C. elegans, drosophila and lower chordates [3-5]. A related gene, presenilin-2 also exists and mutations in this gene also cause familial Alzheimer’s disease [1].

Within cells PS1 protein is located primarily in endoplasmic reticulum and Golgi membranes [6,7]. However some protein is found in endosomes and on the surface of cells as well as in the nuclear membrane and at synaptic sites [8-10]. PS1 influences multiple molecular pathways being best known for its role as a component of the γ-secretase complex [2]. However PS1 also interacts with other proteins in manners that do not involve γ-secretase cleavage such as PS1’s well-studied interaction with β-catenin in which PS1 controls β-catenin stability by favoring its stepwise phosphorylation leading to its degradation [11].

Mice with null mutations in PS1 die within 30 min after birth exhibiting multiple CNS and non-CNS abnormalities [12,13]. One of the most prominent features in the developing brains is a vascular dysgenesis that is associated with multiple intracerebral hemorrhages [12-15]. The molecular and cellular basis for the vascular dysgenesis in PS1−/− mice remains incompletely understood. The extracellular matrix plays a key role in vascular development [16] which led us to hypothesize that components of the extracellular matrix might be altered by the absence of PS1.

Fibronectin is one key component of the extracellular matrix. Many extracellular matrix proteins depend on fibronectin for their incorporation into the matrix [17]. Within the extracellular matrix, fibronectin supports cell adhesion in addition to playing functional roles in regulating growth factor [18] and integrin related signaling [17]. Fibronectin is essential for vascular development and fibronectin null mutations in the mouse lead to embryonic lethality with severe vascular defects [19].

Here we report that PS1−/− endothelial cells contain more fibronectin than wild type cells. We further show that fibronectin accumulates in PS1−/− endothelial cells due to decreased turnover of the fibrillar fibronectin matrix. These studies demonstrate a critical role for PS1 in regulating the formation of the extracellular matrix by endothelial cells and may help to explain the basis for the vascular dysgenesis found in PS1−/− mice.

## Methods

### Genetically modified mice

The PS1−/− mice utilized were those generated by Shen et al. [12]. Genotyping was performed as previously described [20]. Heterozygous mice were mated to produce PS1−/− embryos with the day a vaginal plug was detected designated as E0.5. Pregnant female mice were euthanized with carbon dioxide and PS1−/− embryos were presumptively identified based on their gross dysmorphic appearance. A portion of the body was saved and used to isolate DNA and confirm genotypes. All protocols were approved by the Institutional Animal Care and Use Committee of the James J. Peters Department of Veterans Affairs Medical Center (Bronx, NY USA) and were conducted in conformance with Public Health Service policy on the humane care and use of laboratory animals and the *NIH Guide for the Care and Use of Laboratory Animals*.

### Generation of wild type and PS1−/− endothelial cell cultures

Endothelial cells were prepared from E15.5 to E16.5 embryonic brains. To maximize the yield the entire brain was used to prepare cultures after removal of the meninges. Endothelial cell cultures were prepared as described previously [21]. Cultures were continuously passaged on tissue culture dishes coated with murine collagen type IV (BD Biosciences, Franklin Lakes, NJ, USA) in endothelial cell growth medium (ECGM: DMEM-F12 supplemented with 10% heat-inactivated horse serum, 10% heat-inactivated fetal calf serum, 100 μg/ml endothelial cell growth supplement [BD Biosciences, Franklin Lakes, NJ, USA], and 100 μg/ml heparin). Continuous cell lines were established by continuously passing the cells at high density (1:2 split ratio) in ECGM.

### Preparation of embryonic microvessel fractions

Embryos from PS1−/− and wild type littermate controls were collected at gestational ages ranging from E14.5 to 18.5. Brains were dissected, suspended in phosphate buffered saline (PBS) and mechanically dissociated with a fire-polished Pasteur pipette. The suspension was filtered through a 75 μm nylon mesh filter. The microvessels retained on the filter were extensively washed with cold PBS and collected by centrifugation. To increase yields two to three brains per genotype were pooled from each litter.

### Immunostaining

Endothelial cells were cultured on collagen IV coated slides and fixed with 4% paraformaldehyde/PBS at room temperature or with acetone/methanol (2:3 v/v) at −20°C. Immunostaining was performed as previously described [21] using the following antibodies: a rabbit polyclonal anti-fibronectin (1:400; Sigma Aldrich, St. Louis MO, USA), a rabbit polyclonal antibody against von Willebrand factor (1:400; Sigma Aldrich) and a rat monoclonal anti PECAM/CD31 (1:100; Millipore Billerica, MA USA) followed by appropriate Alexa-conjugated secondary antibodies (Invitrogen, Carlsbad, CA USA). Nuclei were counterstained with 1 μg/ml 4',6-diamidino-2-phenylindole (DAPI). When staining for biotinylated fibronectin, cell cultures were incubated with Alexa conjugated-streptavidin (1:300; Invitrogen). Images were acquired with a Zeiss Axioplan or a Zeiss 700 confocal microscope (Zeiss, Thornwood, NY USA). To quantitate levels of fibronectin expression random fields were photographed under the same exposure with a 20x lens on a Zeiss Axioplan microscope. Images were analyzed with Adobe Photoshop CS4 Extended v11.02 (Adobe Systems Incorporated, San Jose, CA USA) using the analysis tool. Results were expressed as fluorescence intensity/unit area/number of nuclei. Ten random fields containing approximately 100 cells were counted.

For immunohistochemistry E15.5 embryonic brains were collected, fixed overnight in 4% paraformaldehyde/PBS and stored in PBS until sectioning. 50 μm thick sections were cut on a Leica VT1000 Vibratome (Vienna, Austria). Sections were stained with the rabbit polyclonal anti-fibronectin antibody described above (1:400) and with biotin-labeled *Bandeiraea (Griffonia) simplicifolia* lectin (3μg/ml; BSI-B4, Sigma-Aldrich) as previously described [15]. Sections were counterstained with DAPI.

### Endothelial cell electroporation

Endothelial cells were trypsinized, washed with PBS and resuspended in RPMI/10% fetal calf serum (electroporation buffer). 400 μl aliquots containing approximately 3×10^5^ cells were transferred to the electroporation cuvettes (BTX Harvard Apparatus, Holliston, MA USA). Plasmid DNA was added and the mixture chilled 10 min at 4°C. Electroporation was performed with an ECM 830 generator (BTX, Harvard Apparatus) using one 200-volt pulse applied for 40 msec. After a 5 min recovery at room temperature the cells were plated in ECGM. An expression ready plasmid containing human PS1 cDNA was obtained from Genecopeia (Rockville, MD USA).

### Western blot analysis

Cells or embryonic vessel preparations were lysed in a buffer containing 50 mM Tris HCl pH 7.4, 150 mM NaCl, 1 mM EDTA, 1% Triton X-100, 0.5% Na deoxycholate, 0.5% SDS containing protease inhibitors (Halt, Pierce, Rockford IL USA) and phosphatase inhibitor cocktails 2 and 3 (Sigma-Aldrich). After a brief sonication, extracts were centrifuged at 14,000 rpm for 20 min and the supernatants collected. Protein concentration was determined with the BCA reagent as described by the manufacturer (Pierce). Western blotting was performed as previously described [20]. The following antibodies were used: a rabbit polyclonal anti-fibronectin (1:4000; Sigma Aldrich), a rabbit monoclonal anti-vimentin (1:1500, Cell Signaling, Danvers, MA, USA), a mouse monoclonal antibody against the human PS1 N-terminal fragment (NT.1; 1:500; gift of Dr. Paul Mathews, Nathan Kline Institute, Orangeburg NY, USA) and a mouse monoclonal antibody against the PS1 C-terminal fragment (33B10, 1:1000; gift of Dr. Nikolaos Robakis, Icahn School of Medicine at Mount Sinai, New York, NY, USA). A rabbit polyclonal anti β-tubulin (1:5000; Abcam, Cambridge UK) was used as loading control.

### Deoxycholate solubility assay

Deoxycholate (DOC) solubility was assessed as described in Wierzbicka-Patynowski et al. [22]. Endothelial cells were grown in ECGM medium containing fibronectin-depleted serum that had been prepared by chromatography through gelatin-Sepharose [22]. The cells were harvested after 48 hrs, lysed in DOC lysis buffer (2% Na deoxycholate, 20 mM Tris HCl pH 8.8, 2 mM EDTA, 2 mM iodoacetic acid and 2 mM N-ethylmaleimide) and the viscosity reduced by several passages though a 25g needle. The lysate was centrifuged at 14,000 rpm for 30 minutes and the supernatant saved as the DOC soluble fraction. The pellet (i.e. the DOC insoluble fraction) was washed once in DOC lysis buffer, resuspended in lysis buffer containing 1% SDS instead of 2% DOC and boiled for 5 minutes. Protein concentration was determined with the BCA reagent and the fractions were analyzed by Western blotting.

### Fibronectin biotinylation

Purified bovine plasma fibronectin 0.5 mg; Sigma Aldrich was dialyzed against 0.5 M Na carbonate buffer, pH 8.5/0.15 M NaCl overnight at 4°C. NHS-Biotin (Pierce) was added (0.1 mg/ml) and the mixture incubated for 30 min and dialyzed overnight against Tris-buffered saline [23]. Biotinylated fibronectin was added to cells at a concentration of 20 μg/ml. To determine the rate of degradation of exogenously supplied fibronectin endothelial cells were pulsed with biotinylated fibronectin (20 μg/ml) overnight. The cells were then washed with PBS and harvested (0 time point) or switched to FN-depleted ECGM medium and chased for 8 or 24 hrs at which time cells were washed with PBS and the DOC soluble and insoluble fractions were prepared. Samples were analyzed by Western blotting probed with streptavidin-HRP (1:1000; Jackson Immuno Research Laboratories, West Grow, PA USA) for 2 hrs and visualized with the ECL Prime reagent (GE Healthcare).

### Fibronectin labeling and immunoprecipitation

Cells were incubated with Expre^35^S^35^S-protein labeling mix (Perkin-Elmer, Waltham MA, USA) in cysteine/methionine free medium for different time intervals. At each time point the medium was collected and the cells were washed once with PBS and harvested. DOC soluble and insoluble fractions were prepared as described above. The samples were precleared with Agarose beads (Pierce) for 1 hr. Fibronectin was immunoprecipitated by addition of 2.0 μg of anti-fibronectin antibody (see above). After overnight incubation at 4°C, 20 μl of protein A/G slurry (Pierce) was added to capture the immune complexes. The beads were washed 3 times with 25 mM Tris HCl pH 7.4, 0.15 NaCl, 1mM EDTA, 1% NP-40, 5% glycerol and the bound proteins eluted by addition of reducing sample buffer. The eluted proteins were boiled for 10 min and loaded onto a 7.5% SDS-PAGE gel. Gels were fixed for 30 min with isopropanol/water/acetic acid 25/65/10 (v/v/v), and then treated with Amplify reagent (GE Healthcare, Piscatawy, NJ USA) for 30 min, dried and exposed to film.

### RNA isolation and quantitative PCR (qPCR) analysis

Total RNA was isolated using the Rnaeasy kit (Ambion, Austin, TX, USA) according to the manufacturer’s instructions and treated with the DNA free reagent (Ambion) to remove any residual genomic DNA contamination. 0.5-1μg of RNA was reverse transcribed using the High Capacity cDNA reverse transcription kit (Applied Biosystems, Foster City, CA USA). qPCR analysis was performed using predesigned Taqman gene expression assays (Applied Biosystems) for the selected targets as described previously [20]. Normalization was carried out using the geometric means of three genes: peptidylprolyl isomerase A (Ppia), β-glucuronidase (Gusb) and β-actin.

### Statistical procedures

All data are presented as mean ± the standard error of the mean. Equality of variance was assessed using the Levene test. Comparisons were made using unpaired *t* tests (Student’s t if the variances did not differ significantly, p > 0.05, by Levene’s test; otherwise using the Welch correction for unequal variances). Pearson correlations were also utilized. Statistical tests were performed using the program GraphPad Prism 5.0 (GraphPad Software, San Diego, CA USA) or SPSS 20.0 (SPSS, Chicago, IL USA).

## Results

### PS1−/− endothelial cells contain more fibronectin than wild type endothelial cells

**Figure 1:**
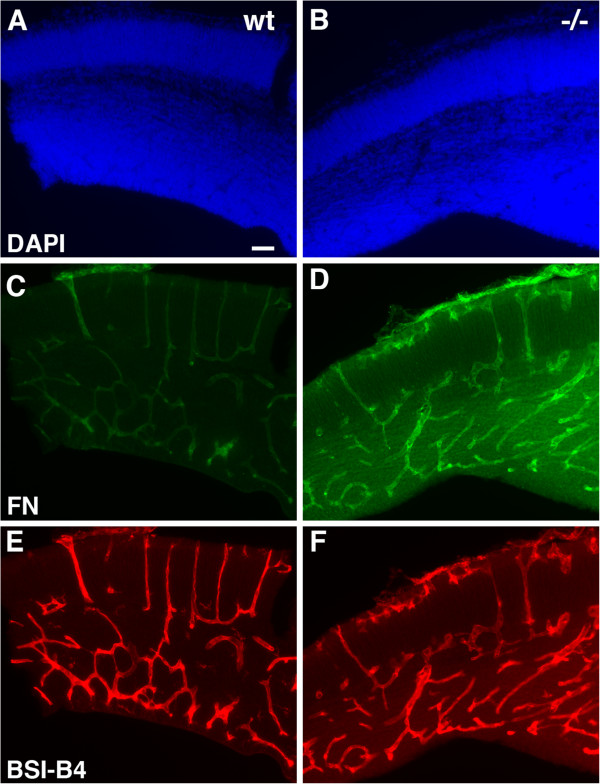
**Increased immunohistochemical staining of blood vessels in PS1−/− embryonic brain with fibronectin.** Shown are neocortical sections from E15.5 wild type (**A**, **C**, **E**) and PS1−/− (**B**, **D**, **F**) embryonic brain double stained with anti-fibronectin (FN) antibodies (**C**, **D**) and the *Bandeiraea (Griffonia) simplicifolia* (BSI-B4) lectin (**E**, **F**). Panels **A** and **B** show DAPI staining of the same sections. Matched sections were photographed under the same conditions. Note the increased vascular staining in the PS1−/− embryo with fibronectin compared to the lectin staining which is equally intense in the wild type and PS1−/− embryo. Scale bar 50 μm.

One of the most prominent features in the brains of PS1−/− embryos is the appearance of parenchymal hemorrhages. Associated with the vascular hemorrhages there is a vascular dysgenesis [15]. In preliminary studies aimed at examining whether components of the extracellular matrix might be altered in PS1−/− mice we noted that developing blood vessels in PS1−/− embryonic brain stained more prominently with fibronectin than wild type embryos while vessels in wild type and PS1−/− brain were visualized equally by the isolectin B4 (Figure 1).

**Figure 2:**
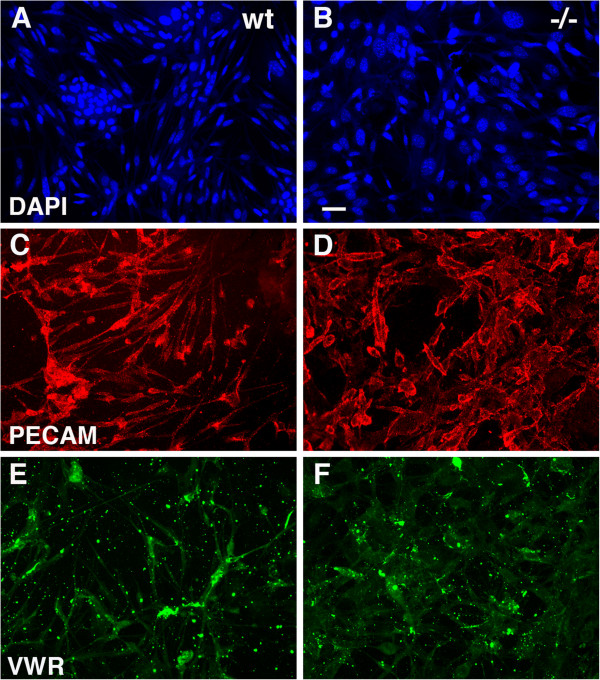
**Characterization of wild type and PS1−/− endothelial cells by immunostaining.** Shown are primary cultures of wild type (**A**, **C**, **E**) and PS1−/− endothelial cells (**B**, **D**, **F**) double stained for PECAM (**C**, **D**) and von Willebrand factor (**E**, **F**). Panels **A** and **B** show the same fields stained for DAPI. Scale bar 20 μm.

**Figure 3:**
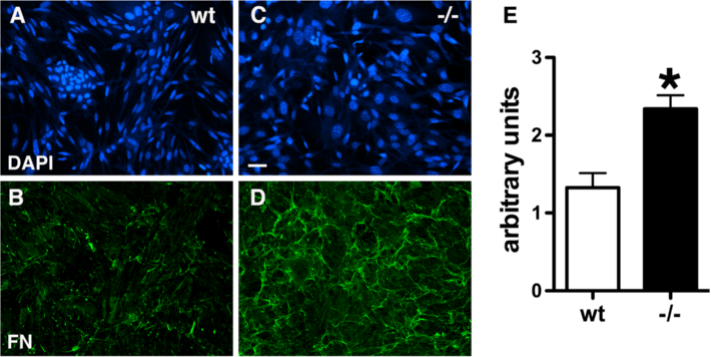
**Increased expression of fibronectin in PS1−/− endothelial cells by immunostaining.** Shown are primary cultures of wild type (**A**, **B**) and PS1−/− endothelial cells (**C**, **D**) immunostained for fibronectin (**B**, **D**). Panels **A** and **C** show the same fields stained for DAPI. In panel **E**, fluorescence intensity of fibronectin staining was measured in wild type and PS1−/− cultures. Data are presented as fluorescence intensity/unit area per number of nuclei counted which was acquired in ten random fields per culture. Asterisk indicates p < 0.05 vs. wild type (unpaired t-test). Scale bar 20 μm.

To determine whether the increased fibronectin expression might reflect a primary overproduction of fibronectin by PS1−/− endothelial cells, we examined fibronectin expression in endothelial cells cultured from wild type and PS1−/− embryos. Primary endothelial cells were isolated from E15.5-E16.5 brain using a procedure that we previously developed [21]. Characterization of the cells by immunostaining showed that both wild type and PS1−/− endothelial cells expressed the endothelial cell markers PECAM-1 and von Willebrand factor (Figure 2). Cultures from both wild type and PS1−/− embryos established apparent continuous cell lines that have now been passaged over 40 times and still retain their endothelial cell character as indicated by the expression of PECAM-1 (data not shown). Immunostaining for fibronectin showed that PS1−/− endothelial cells had increased levels of fibronectin as compared to wild type cells (Figure 3).

**Figure 4:**
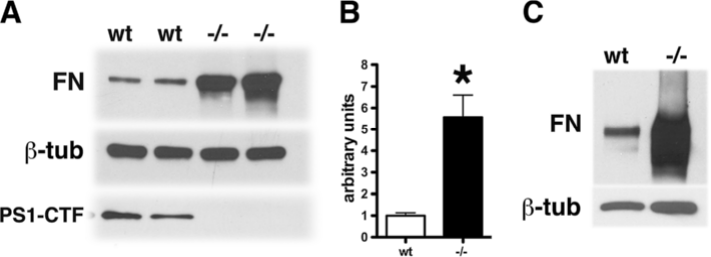
**Increased levels of fibronectin in PS1−/− endothelial cells by Western blotting.** Primary cultures of wild type and PS1−/− endothelial cells were grown to confluency in complete growth media containing fibronectin-depleted serum. The cells were harvested after washing with PBS and total cell lysates were prepared. Western blotting was performed using an anti-fibronectin (FN) antibody (upper panel in **A**). The middle panel shows the blot reprobed for β-tubulin (β-tub) as a loading control. The lowest panel in **A** shows the extracts probed with the antibody 33B10 which recognizes the C-terminal fragment (CTF) of PS1 to confirm the lack of detectible PS1 expression in PS1−/− endothelial cells. Panel **B** shows quantitation of the levels of fibronectin in the experiment shown in panel **A**. Asterisk indicates p < 0.05 vs. wild type (unpaired t-test). In panel **C**, passage 38 wild type or PS1−/− endothelial cells were cultured as above overnight. Total cell lysates were prepared and Western blotting was performed using an anti-fibronectin antibody (upper panel). Lower panel shows the blot reprobed for β-tubulin. All lanes were loaded with 10 μg of total protein. Representative blots are shown from experiments that were independently replicated three times.

To examine fibronectin expression biochemically we performed Western blotting on primary cultures of wild type and PS1−/− endothelial cells. Total cell lysates were prepared from cultures grown to confluency in fibronectin depleted growth media. Western blot analysis showed that total fibronectin was elevated in primary cultures of PS1−/− endothelial cells 2–6 fold depending on the preparation. A representative blot from three independent experiments is shown in Figure 4A,B. Fibronectin was also increased in continuously passaged cell lines of endothelial cells from PS1−/− embryos (Figure 4C).

**Figure 5:**
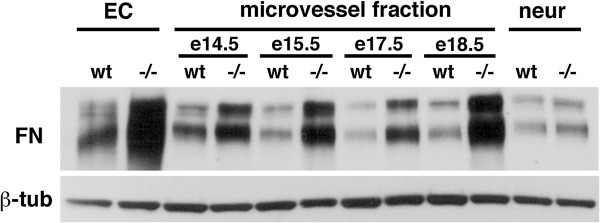
**Fibronectin levels are increased in microvascular preparations from PS1−/− embryonic brain.** Microvascular preparations were collected on nylon filters from brains of the indicated embryonic (e) ages. “neur” indicates the “neuronal” fraction that passed through the nylon filer which was collected by centrifugation and then lysed for blotting. Blots were sequentially probed for fibronectin (FN) and β-tubulin (β-tub). For comparison primary endothelial cell cultures (EC) were prepared and allowed to expand for two weeks before being cultured in serum free growth media overnight and then harvested. 10 μg of protein was loaded per lane. A representative blot is shown from experiments that were performed three times.

Because it remained possible that the culturing of endothelial cells was affecting the expression of fibronectin we examined fibronectin expression in microvascular preparations captured on nylon membranes from wild type and PS−/− embryos. As shown in Figure 5, fibronectin levels were increased in PS1−/− microvascular preparations from E14.5 to E18.5 embryonic brain. By contrast fibronectin levels were low and showed no difference between wild type and PS1−/− in the neuronal fraction that passed through the nylon filter from embryos collected at E 15.5.

**Figure 6:**
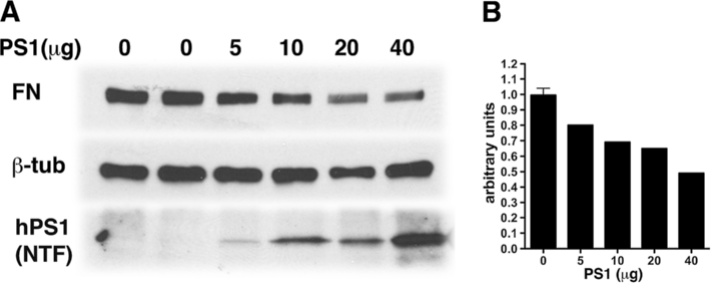
**Reintroduction of PS1 into PS1−/− endothelial cells leads to decreased fibronectin expression.** PS1−/− endothelial cells (p41), grown to confluency were trypsinized, resuspended in electroporation buffer and electroporated with a plasmid carrying the human PS1 cDNA or empty vector. In panel **A**, the expression of fibronectin was analyzed 48 hrs post-electroporation by Western blotting on cell lysates. 10 μg of protein was loaded per lane. Blots were sequentially probed for fibronectin (FN), and β-tubulin (β-tub). The expression of PS1 was determined with an antibody (NT.1) that is specific for the human (hPS1) N-terminal fragment of PS1 (NTF). The amount of electroporated cDNA (μg) is indicated above each lane. Mock-transfected cells were electroporated with 40 μg of empty vector. In panel **B**, the experiment in panel **A** is quantitated. A Pearson correlation coefficient showed that there was a significant negative correlation between the amount of PS1 plasmid transfected and the level of expression of fibronectin (R^2^=0.8584; p=0.0237). This experiment was replicated twice using transfections of 20 μg of PS1 cDNA or empty vector.

While clonal effects should not be an issue in primary cell cultures, to show that the effects in passaged PS1−/− endothelial cells was due to the loss of PS1 we reintroduced human PS1 into PS1−/− endothelial cells using electroporation. Cells were electroporated with different amounts of PS1 cDNA up to 40 μg and fibronectin expression was examined 48 hrs post-transfection. As shown in Figure 6, progressively higher levels of PS1 expression led to a progressive decrease in the level of fibronectin expression consistent with the overexpression of fibronectin in PS1−/− endothelial cells being due to the loss of PS1. This experiment was independently replicated twice using transfections of 20 μg of PS1 cDNA or empty vector (data not shown). Collectively these results suggest that lack of PS1 was associated with the increased fibronectin.

### Levels of fibronectin RNA in PS1−/− endothelial cells

**Figure 7:**
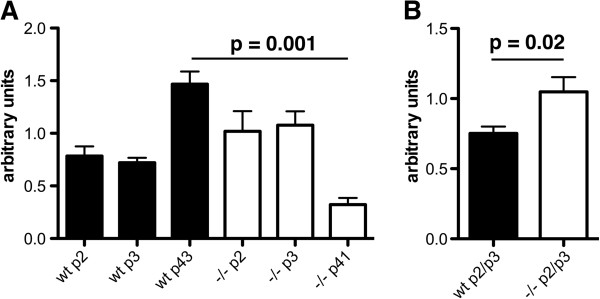
**Levels of fibronectin RNA are not consistently changed in PS1−/− endothelial cells.** In panel **A**, fibronectin RNA was measured in endothelial cell cultures (n=3/genotype per passage) by qPCR for the passages indicated. Samples were run in triplicate and normalized to the geometric means of Ppia, β-actin and Gusb. In panel **B**, the pooled data for passages 2 and 3 is presented. Statistically significant differences (unpaired t-tests) are indicated above selected comparisons. Statistical comparisons are discussed further in the text.

Because increased fibronectin RNA levels might contribute to increases in fibronectin protein we determined fibronectin RNA levels in wild type and PS1−/− endothelial cells by qPCR. Figure 7 shows fibronectin RNA levels in both early and late passage endothelial cells. In early passage (p2/p3) endothelial cells fibronectin RNA was increased about 50% in PS1−/− compared to wild type endothelial cells (p = 0.02, unpaired t-test). With time in culture fibronectin RNA approximately doubled in wild type endothelial cells between early passage (2/3) and passage 43 (p = 0.0003). By contrast in PS1−/− endothelial cells with time in culture fibronectin RNA levels fell by over 50% between early passage (2/3) and passage 41 (p = 0.002), this despite increased levels of fibronectin protein in later passaged cells (Figure 4). p41 PS1−/− endothelial cells also had less that 1/3d the level of fibronectin RNA found in p43 wild type endothelial cells (p = 0.001), this despite fibronectin protein levels being increased in later passaged (p38) PS1−/− compared to wild type endothelial cells (Figure 4C). Thus changes in fibronectin RNA levels correlate poorly with changes in fibronectin protein levels with time in culture and cannot explain the increase in fibronectin protein in PS1−/− endothelial cells.

### More fibrillar fibronectin is present in PS1−/− endothelial cells

**Figure 8:**
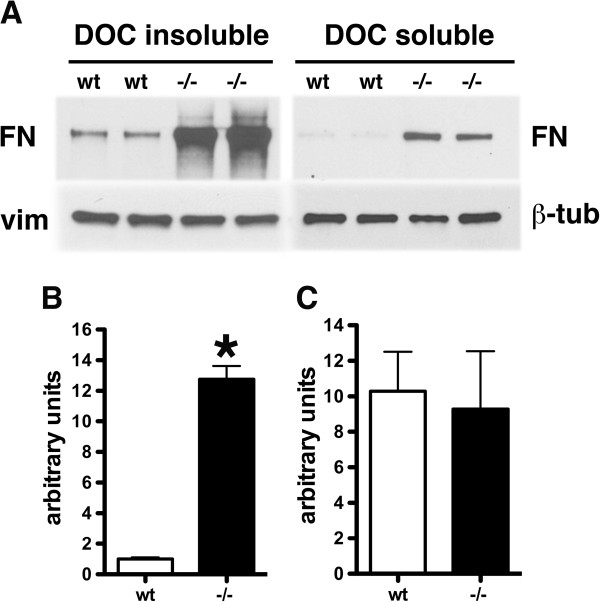
**The fibrillar state of fibronectin is increased in PS1−/− endothelial cells.** Passage 3 wild type and PS1−/− endothelial cells were grown to confluency in fibronectin-depleted medium. The endothelial cells were lysed with deoxycholate (DOC) lysis buffer and DOC-insoluble material was isolated by centrifugation and analyzed by Western blot. In panel **A**, blots of DOC-insoluble and soluble material were sequentially probed for fibronectin (FN) and vimentin (vim) or fibronectin and β-tubulin (β-tub). Panel **B** shows quantitation of the levels of DOC insoluble fibronectin in the experiment shown in panel **A**. In panel **C**, the ratio of DOC insoluble/DOC soluble fibronectin is shown. Asterisk indicates p < 0.05 vs. wild type (unpaired t-test). 1 μg of protein was loaded per lane. A representative blot is shown from experiments that were performed twice.

When secreted fibronectin binds to cells, dimeric fibronectin is converted into a complex network of fibrils that consist of high molecular weight aggregates. The fibronectin found in PS1−/− endothelial cells appeared fibrillar based on the pattern of immunocytochemical staining (Figure 3). The fibrillar state of fibronectin can also be monitored by isolation and quantitation of the relative amounts of deoxycholate (DOC) soluble and insoluble material [24,25]. Therefore we determined relative amounts of fibronectin in DOC soluble and insoluble fractions as a measure of fibrillar state. DOC soluble and insoluble fractions were isolated from wild type and PS1−/− endothelial cells (p3) grown to confluency in fibronectin depleted medium and analyzed by Western blotting. Figure 8 shows a representative blot from two independent experiments. Both DOC soluble and insoluble material were increased in PS1−/− endothelial cells. The ratio of DOC insoluble/DOC soluble fibronectin was also similar in wild type and PS1−/− cells (Figure 8C) indicating that the excess fibronectin produced in PS1−/− endothelial cells is incorporated into a fibrillar network.

### Synthesis of fibronectin is not increased in PS1−/− endothelial cells

**Figure 9:**
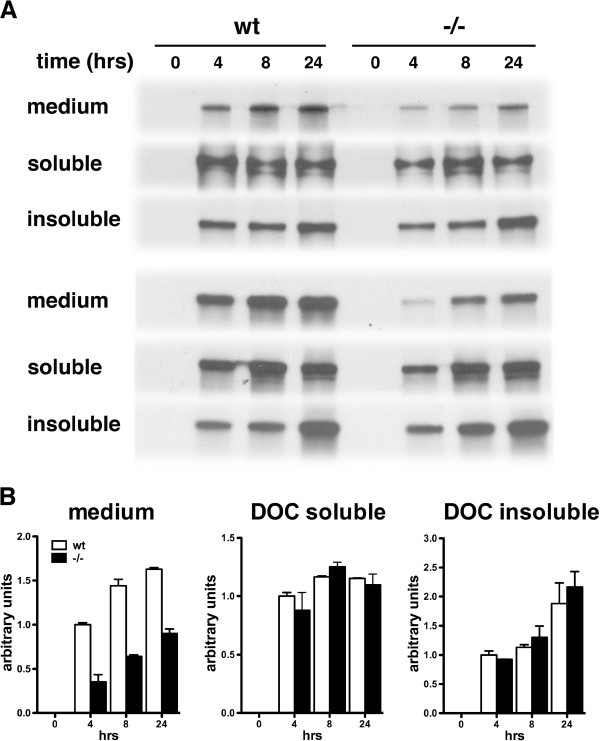
**Lack of increased synthesis of fibronectin in PS1−/− endothelial cells.** p43 wild type (wt) and p41 PS1−/− endothelial cells were metabolically labeled with ^35^S cysteine/methionine. Fibronectin was immunoprecipitated from the culture medium as well as in the DOC soluble and insoluble cellular fractions and levels were determined by fluorography. In panel **A**, duplicate experiments are shown where fibronectin was measured after labeling for the indicated times (hours). In panel **B**, the results of the experiments in panel **A** are quantitated.

Increased levels of fibronectin in PS1−/− endothelial cells could reflect increased synthesis in the absence of PS1. We determined the rate of fibronectin synthesis in wild type and PS1−/− endothelial cells using metabolic labeling. Endothelial cell proteins were labeled with ^35^S cysteine/methionine for from 4 to 24 hours. At chosen time points the amount of fibronectin was measured in the culture medium as well as in the DOC soluble and insoluble cellular fractions. As shown in Figure 9, levels of fibronectin in the medium and DOC insoluble fraction progressively increased from 4 to 24 hours in both wild type and PS1−/− cultures while the level of DOC soluble fibronectin remained constant from 4 to 24 hours. However levels of fibronectin in the medium of PS1−/− endothelial cell cultures were approximately 50% of those found in wild type cultures at each time point while there was no difference between PS1−/− and wild type cultures in the levels of fibronectin in the DOC soluble or insoluble fractions. Thus these studies provide no evidence for any increased fibronectin synthesis in PS1−/− endothelial cells.

### Increased assembly of a fibronectin matrix on the surface of PS1−/− endothelial cells

**Figure 10:**
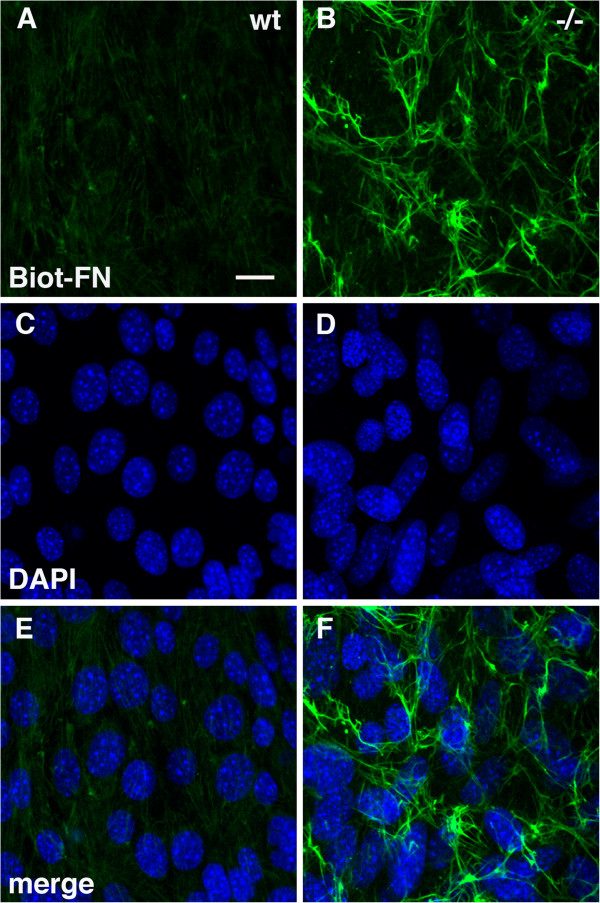
**Increased binding of biotinylated fibronectin to PS1−/− endothelial cells.** p43 wild type (**A**, **C**, **E**) and p41 PS1−/− (**B**, **D**, **F**) endothelial cells were loaded overnight with 20 μg/ml of biotinylated fibronectin. Cells were fixed and stained with Alexa488-conjugated streptavidin and then imaged by confocal microscopy. Panels **A** and **B** show cultures labeled for biotinylated fibronectin. Panels **C** and **D** show the same fields stained for DAPI and merged images are shown in panels **E** and **F**. Scale bar 10 μm.

The above studies suggested that increased levels of fibronectin in PS1−/− endothelial cells could not by explained by increased synthesis. Rather they suggested that PS1 might be influencing fibronectin matrix assembly and maturation. Fibronectin matrix assembly begins with secretion of soluble fibronectin dimers that associate into fibrils and then a fibrillar network [17,26]. This process can be monitored by following the processing of exogenously added fibronectin to cells. Wild type and PS1−/− endothelial cells were loaded overnight with biotinylated fibronectin. Binding was firstly assessed by staining cells with fluorescently labeled streptavidin. As shown in Figure 10, after an overnight exposure PS1−/− endothelial cells contained grossly more biotinylated fibronectin bound to the cell surface that appeared to be assembled into a fibrillar network.

**Figure 11:**
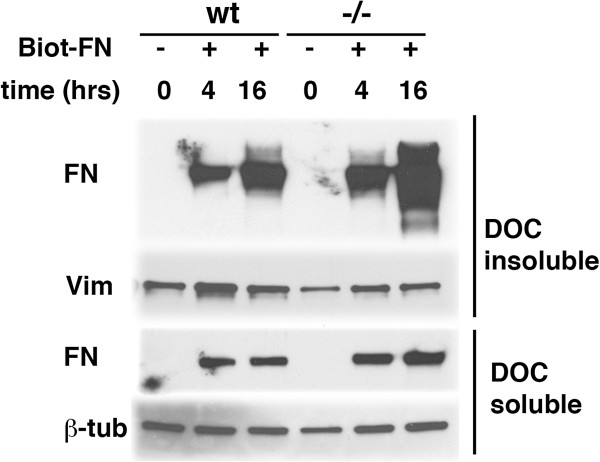
**Increased levels of DOC insoluble biotinylated fibronectin following loading of PS1−/− endothelial cells.** Passage 2 cultures of wild type (wt) and PS1−/− endothelial cells were grown in fibronectin-depleted serum and were loaded with biotinylated fibronectin (Biot-FN: 20 μg/ml) for the indicated times (hours). DOC-soluble and insoluble fractions were prepared and analyzed by Western blot. The membranes were probed with peroxidase conjugated-streptavidin and the DOC insoluble fraction was reprobed for vimentin (vim) while the DOC soluble fraction was reprobed for β-tubulin (β-tub). 1 μg of protein was loaded per lane.

To determine whether the bound fibronectin was assembled into a fibrillar network biochemically, DOC soluble and insoluble fractions were prepared and analyzed by Western blotting. As shown in Figure 11, biotinylated fibronectin was proportionately increased in both DOC-soluble and insoluble fractions isolated from PS1−/− endothelial cells suggesting that more fibronectin was being held in an assembled matrix on the cell surface of PS1−/− endothelial cells.

### Turnover of biotinylated fibronectin is reduced in PS1−/− endothelial cells

**Figure 12:**
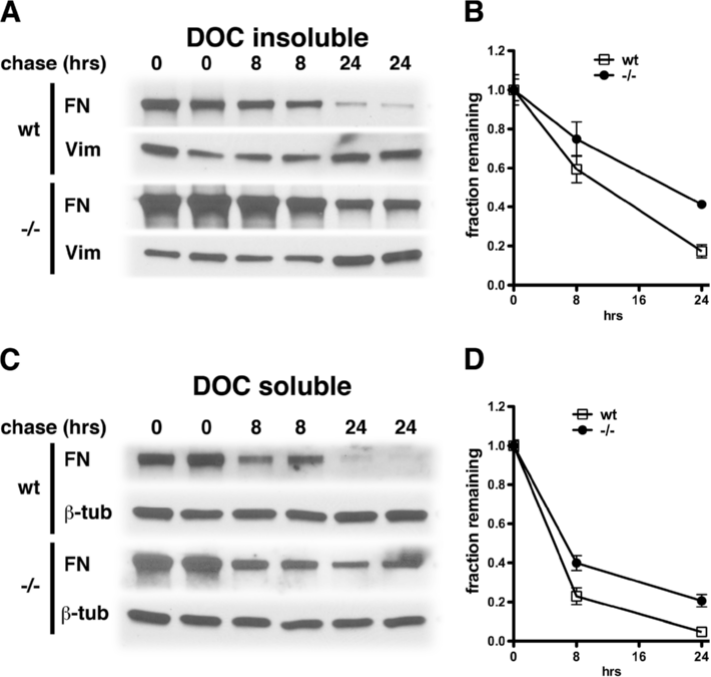
**Delayed turnover of the fibronectin matrix in PS1−/− endothelial cells.** Endothelial cells (p44) were pulsed with biotinylated fibronectin overnight and then switched to FN depleted medium and chased for the indicated times (hrs). At each time point the DOC soluble and insoluble fractions were prepared. One μg (insoluble) or 4 μg (soluble) samples were analyzed for biotinylated-FN by Western blotting using streptavidin-HRP. Samples were probed for vimentin (vim) and β-tubulin (β-tub) by Western blotting as controls for extraction of the insoluble and soluble fractions. Two independent replicates are shown.

The above studies suggested that rather than affecting synthetic rate, the absence of PS1 was affecting the rate of fibronectin turnover on the cell surface. To determine the turnover rate of fibronectin on the cell surface, wild type and PS1−/− endothelial cells were loaded overnight with biotinylated fibronectin and the relative amounts of fibronectin in the DOC soluble and insoluble fractions were determined at 0, 8 and 24 hours after labeling. As shown in Figure 12, biotinylated fibronectin disappeared from PS1−/− cells more slowly than in endothelial cells containing PS1. These results indicate that decreased turnover of the fibronectin matrix best explains the increased levels of fibronectin found in PS1−/− endothelial cells.

## Discussion

Fibronectin is a modular protein that is derived from a single gene which can be alternatively spliced into 20 possible monomeric forms in man and up to 12 in mouse [17]. Fibronectin exists in a cellular form that is present in tissues and assembled into a fibrillar matrix, as well as a plasma form that is produced by the liver and secreted into the blood where it is soluble and nonfibrillar [26]. The assembled fibronectin matrix binds other components of the extracellular matrix [27]. Within the extracellular matrix, fibronectin supports cell adhesion but plays other functional roles as well such as its role in regulating activation of latent complexes containing the transforming growth factor-β [18,28].

Here we show that PS1 regulates fibronectin levels in endothelial cells by modulating the constitutive turnover of the fibronectin matrix. PS1−/− endothelial cells contained more fibronectin protein that assembled into a fibrillar network. The increased fibronectin protein could not be explained by altered levels of fibronectin RNA nor by increased protein synthesis. Rather the increased fibronectin in PS1−/− endothelial cells resulted from a slower rate of degradation of the fibrillar fibronectin matrix assembled on the cell surface.

Fibronectin matrix assembly begins with secretion of soluble fibronectin dimers ( reviewed in [17] ) that bind to integrin receptors on the cell surface. Then in a process that is still incompletely understood fibronectin to fibronectin associations occur that lead to fibril formation and production of a fibrillar network [17]. Integrins are a family of cell surface receptors composed of non-covalently linked heterodimers composed of α and β subunits [29,30]. Integrin α5β1 is the major fibronectin receptor although other integrins can perform this function in some circumstances [31,32]. Integrins signal through a dynamic spatially and temporally controlled process that involves assembly of multiprotein complexes through their cytoplasmic tails [33].

Following fibronectin binding to an integrin receptor, bound fibronectin is first diffusely localized on the cell surface [17]. Fibronectin binding promotes receptor clustering and the dimeric fibronectin becomes organized into short fibrils that are initially DOC soluble [17]. Thin fibrils lengthen and are converted into a DOC insoluble form.

Integrin receptor activation further leads to the cytoplasmic domains of integrins becoming associated with and activating the cytoskeleton. During this process complexes containing α5β1 integrin, focal adhesion kinase (FAK), vinculin, and paxillin form at sites of fibronectin fibril assembly leading to activation of FAK, the recruitment and activation of Src family kinases and activation of the phosphoinositide 3-kinase (PI3K) pathway among others [33,34].

Where PS1 is acting in the molecular events that regulate fibronectin matrix turnover is unclear. PS1 has been reported to affect maturation of the β1 integrin subunit in fibroblasts [35]. However, in exploring levels of integrins in PS1−/− endothelial cells we have not found any consistent changes in levels of the α5 or β1 subunits (unpublished observations). Functionally PS1 is best known for its role as a component of the γ-secretase complex which is known to cleave more than 60 transmembrane proteins [2]. Therefore PS1 could be regulating fibronectin turnover if it affected signaling through integrin receptors. Integrins are transmembrane proteins made of α/β heterodimers with each subunit having a large extracellular domain, a single transmembrane helix and a short cytoplasmic segment [36]. There is currently no evidence that integrin subunits are cleaved by γ-secretase. However, PS1 influences some of the known pathways regulated by integrin signaling. For example PS1 has been reported to enhance signaling through the PI3K/Akt pathway by associating with the p85 regulatory subunit of PI3K [37,38] although why this interaction should affect turnover of the fibronectin matrix is unclear. Fibronectin matrix turnover also occurs through a caveolin-1 dependent process [39] and caveolin-1 dependent trafficking has been reported to be affected by the absence of PS1 [40] providing another mechanism whereby PS1 could affect fibronectin matrix remodeling.

## Conclusions

Future studies will be needed to determine how PS1 affects remodeling of the fibronectin matrix at the cell surface. However whatever its mechanism of action, these studies show that PS1 is essential for the constitutive remodeling of the fibronectin matrix in endothelial cells. The extracellular matrix plays crucial roles in the development and function of the cerebral vasculature [16] and fibronectin is essential for normal vasculogenesis with the absence of fibronectin leading to severe embryonic vascular defects [19]. Whether ineffective remodeling of the fibronectin matrix may help to explain the vascular dysgenesis in the brains of PS1−/− embryos is as yet unclear. However, these studies suggest molecular clues to the origins of the vascular dysgenesis found in PS1−/− embryonic mice that can be explored in future studies.

## Abbreviations

β-tub: β-tubulin; cDNA: complementary DNA; CNS: central nervous system; CTF: C terminal fragment; DAPI: 4',6-diamidino-2-phenylindole; DOC: deoxycholate; DMEM/F12: Dulbecco's Modified Eagle Medium/Ham’s F12; ECGM: endothelial cell growth medium; EDTA: ethylenediaminetetraacetic acid; FN: fibronectin; kDa: kilo Dalton; Gusb: β-glucuronidase; NP-40: Nonidet-P40 (octyl phenoxylpolyethoxylethanol); NTF: N terminal fragment; PI3K: phosphoinositide 3 kinase; PBS: phosphate buffered saline; PCR: polymerase chain reaction; PECAM: platelet endothelial cell adhesion molecule; Ppia: peptidylprolyl isomerase A; PS1: presenilin-1; qPCR: quantitative polymerase chain reaction; RPMI: Roswell Park Memorial Institute medium; SDS-PAGE: sodium dodecyl sulfate polyacrylamide gel electrophoresis; vim: vimentin; Wt: wild type.

## Competing interests

The authors have no competing personal, financial or non-financial interests.

## Authors’ contributions

RDG participated in the design and execution of all the experiments described in this paper as well as participated in the manuscript writing; MAGS participated in the experimental design as well as generation and maintenance of the endothelial cell lines and manuscript writing; GAE participated in the experimental design, data analysis and manuscript writing. All authors read and approved the final manuscript.
